# *EGFR* and *ERBB2* exon 20 insertion/duplication in advanced non–small cell lung cancer: genomic profiling and clinicopathologic features

**DOI:** 10.3389/fonc.2023.1163485

**Published:** 2023-05-22

**Authors:** Ramakrishna R. Sompallae, Bilge Dundar, Natalya V. Guseva, Aaron D. Bossler, Deqin Ma

**Affiliations:** Department of Pathology, University of Iowa Hospitals & Clinics, Iowa City, IA, United States

**Keywords:** *EGFR/ERBB2* exon20 insertion/duplication, non-small cell lung cancer, ERBB2, clinicopathologic features, PD-L1

## Abstract

**Background:**

Exon 20 (ex20) in-frame insertions or duplications (ins/dup) in epidermal growth factor receptor (*EGFR*) and its analog erb-b2 receptor tyrosine kinase 2 (*ERBB2*) are each detected in 1.5% of non–small cell lung cancer (NSCLC). Unlike *EGFR* p.L858R or ex19 deletions, ex20 ins/dup is associated with *de novo* resistance to classic EGFR inhibitors, lack of response to immune checkpoint inhibitors, and poor prognosis. US Food and Drug Administration has approved mobocertinib and amivantamab for targeting tumors with this aberration, but the number of comprehensive studies on ex20 ins/dup NSCLC is limited. We identified 18 cases of NSCLCs with *EGFR/ERBB2* ex20 ins/dup and correlated the findings with clinical and morphologic information including programed death-ligand 1 (PD-L1) expression.

**Methods:**

A total of 536 NSCLC cases tested at our institution between 2014 and 2023 were reviewed. A custom-designed 214-gene next-generation sequencing panel was used for detecting DNA variants, and the FusionPlex CTL panel (ArcherDx) was used for the detection of fusion transcripts from formalin-fixed, paraffin-embedded tissue. Immunohistochemistry (IHC)for PD-L1 was performed using 22C3 or E1L3N clones.

**Results:**

Nine *EGFR* and nine *ERBB2* ex20 ins/dup variants were identified from an equal number of men and women, 14 were non- or light smokers, and 15 had stage IV disease. All 18 cases were adenocarcinomas. Seven of the 11 cases with available primary tumors had acinar predominant pattern, two had lepidic predominant pattern, and the remainder had papillary (one case) and mucinous (one case) patterns. Ex20 ins/dup variants were heterogenous in-frame one to four amino acids spanning A767–V774 in *EGFR* and Y772–P780 in *ERBB2* and were clustered in the loop following the C-helix and α C-helix. Twelve cases (67%) had co-existing *TP53* variants. Copy number variation in *CDK4* amplification was identified in one case. No fusion or microsatellite instability was identified in any case. PD-L1 was positive in two cases, low positive in four cases, and negative in 11 cases.

**Conclusions:**

NSCLCs harboring *EGFR/ERBB2* ex20 ins/dup are rare and tend to be acinar predominant, negative for PD-L1, more frequent in non- or light smokers, and mutually exclusive with other driver mutations in NSCLC. The correlation of different *EGFR/ERBB2* ex20 ins/dup variants and co-existing mutations with response to targeted therapy and the possibility of developing resistant mutations after mobocertinib treatment warrants further investigation.

## 1 Introduction

Lung cancer is the second most common cancer in both men and women and the leading cause of cancer death, accounting for about one in five of all cancer deaths in the United States (https://www.cancer.org/cancer/lung-cancer/about/key-statistics.html). Non–small cell lung cancer (NSCLC) accounts for 82% of lung cancer. In patients with lung adenocarcinoma, epidermal growth factor receptor (*EGFR*) mutations are present in 10%–20% of Caucasian and in 40%–60% of South-East Asian population (1). Up to 90% *EGFR* mutations are exon 19 in-frame deletions or the p.L858R hotspot mutation in exon 21 (1). Ex20 insertion/duplication (ins/dup) is the third most common *EGFR* mutation and detected in approximately 1.5% of NSCLC (2–5).

Erb-b2 receptor tyrosine kinase 2 (ERBB2) encodes for ERBB2, also called human epidermal growth factor receptor 2 (HER2), which is another member of the ERBB family of receptor tyrosine kinases. *EGFR* and erb-b2 receptor tyrosine kinase 2 (ERBB2) share structural and sequence similarities. Both have an extracellular ligand-binding domain, a transmembrane domain, and a tyrosine kinase domain (6). *ERBB2* ex20 ins/dup has also been identified in NSCLCs and shows a similar mutation frequency of 1.5% (3, 7).

Previous studies have shown that similar to other *EGFR* variants, *EGFR/ERBB2* ex20 ins/dup is predominantly found in adenocarcinoma, non-smokers, and women (1). In comparison to the more common and other uncommon *EGFR* mutations, ex20 ins/dup was associated with poor prognosis and lower overall survival (OS) (8).

Unlike tumors with *EGFR* p.L858R and ex19 deletion that are sensitive to first- and second-generation tyrosine kinase inhibitors (TKIs), ex20 ins/dup has *de novo* resistance to classic *EGFR* TKIs including the third-generation TKI such as osimertinib that targets the p.T790M in ex20 of the *EGFR* (9–11) and no response to immune checkpoint inhibitors (ICIs) (4). The US Food and Drug Administration (FDA) approved mobocertinib and amivantamab that specifically target *EGFR* and *ERBB2* ex20 ins/dup. Targeted therapy for patients with this aberration showed sustained response (12, 13).

Because of the low incidence of the ex20 ins/dup in NSCLC, there have been a limited number of comprehensive studies published, and about half of the existing studies were performed in the Asian population with a focus on *EGFR* ex20 ins/dup only. In this study, we identified 18 cases of NSCLCs with *EGFR/ERBB2* ex20 ins/dup (nine cases each) from 536 cases tested at our institution using next-generation sequencing (NGS) assays and performed comprehensive molecular profiling of these cases. The molecular findings were correlated with clinical information and morphology. PD-L1 expression and survival data were also evaluated.

## 2 Methods

### 2.1 Case selection

This study was approved by the Institutional Review Board. Pathology archives were searched for NSCLC cases that had undergone NGS testing between July 2014 and January 2023. Five hundred thirty-six cases were identified, of which 18 were positive for either *EGFR* or *ERRB2* ex20 ins/dup. For *EGFR/ERBB2* ex20 ins/dup-positive cases, additional clinical information was obtained by chart review including patient’s age, gender, smoking history, histologic subtypes, tumor stage, PD-L1 status, progression-free survival (PFS) (time from diagnosis to first metastasis), treatment received, and survival time after initial diagnosis.

Light smokers are those with a smoking history of 1 to 20 packs per year.

### 2.2 Extraction of nucleic acid

Each case was reviewed by two pathologists, and the optimal formalin-fixed, paraffin-embedded (FFPE) block was selected for testing. The minimal percentage of tumor content was 10%. One hematoxylin and eosin (H&E)–stained slide along with 10 unstained sections (6 µm in thickness) was cut. Areas of interest were circled on the H&E slide, and corresponding areas from the unstained slides were manually scraped using a razor blade. After deparaffinization with xylene and ethanol wash of the pellet, the total nucleic acid was extracted using the RNeasy FFPE mini kit (Qiagen, Valencia, CA) excluding the DNAase treatment step. The concentration of the DNA and RNA was determined using Qubit 2.0 fluorometer (Thermo Fisher Scientific, Waltham, MA).

### 2.3 Next-generation sequencing analysis

A custom-designed DNA-based 214-gene NGS panel that covers the full coding sequence of 94 genes and hotspot regions of 120 genes was used for the detection of single-nucleotide variant (SNV), small deletion/duplication, copy number variants (CNV) in 49 genes, and microsatellite instability (MSI) status. DNA (40 ng) was used to generate NGS libraries, and sequencing was performed on the NextSeq (Illumina, Inc., San Diego, CA). Data were analyzed using the Burrows-Wheeler Aligner (BWA) and the Pisces variant caller v.2.1. This assay has a limit of detection of 2.5% for SNV and 6.8% for insertions/deletions. A copy number ratio of 1.9 and 0.5 combined with a z-score ≥ 5.0 was considered as a gene copy gain and loss, respectively.

The RNA-based Comprehensive Thyroid and Lung (CTL) FusionPlex Assay (IDT Technologies, Inc., Coralville, IA) was used for the detection of gene fusions following the manufacturer’s protocol. Briefly, total RNA (250 ng) was reverse-transcribed to cDNA, which was subsequently processed with end repair and dA tailing, followed by ligation with a half-functional adapter that allows amplification from the gene-specific primer (GSP) in one direction only. Two rounds of PCR were performed using GSPs for target enrichment. Libraries were pooled, typically eight at a time, in equimolar concentrations and sequenced using the MiSeq instrument (Illumina Inc., San Diego, CA). A denatured PhiX library was added to each run as a sequencing quality control. The sequence data were analyzed using the CTL Target Region File and vendor-supplied software (Archer Analysis versions 5.0 and 6.0). A minimum of five reads with three or more unique sequencing start sites that cross the breakpoints were set as the cutoff to call for strong evidence of fusions. The Target Mutation file (a text file in variant caller format (VCF) that lists the specific variants of interest) was created and used for targeted variant analysis.

### 2.4 Immunohistochemistry studies for PD-L1

PD-L1 immunohistochemistry (IHC) study was performed using the standard protocols in the clinical laboratory. Slides (3 μm in thickness) were deparaffinized, followed by rehydration, and heat-induced epitope retrieval in an ethylenediaminetetraacetic acid (EDTA) buffer at pH 9.0. Antibody 22C3 or E1L3N clones were used. Tumor proportion score (TPS) and immune cell (IC) staining score were used for 22C3 clone, and tumor cell (TC) (4) and IC staining score were used for the E1L3N clone. For TCs and TPSs, >1% was considered low positive, ≥50% was considered positive, and ≥5% tumor area occupied by ICs was considered positive.

## 3 Results

### 3.1 Patients characteristics

A total of 536 NSCLCs were tested; among them 18 (3.3%) harbored *EGFR* or *ERBB2* ex20 ins/dup. There were an equal number of men and women. The mean age at the time of diagnosis was 63 years and ranged from 41 to 83 years. There were 15 Caucasian (88%, n = 17), two African Americans, and one patient with unknown ethnicity. Fourteen patients were never (nine) or light (five) smokers, three were heavy smokers, and one had an unknown smoking history. Among the 15 patients with available treatment information, seven received chemoradiation therapy; one each had chemotherapy or radiation therapy only, and one did not receive any treatment. Six patients received pembrolizumab: five with chemotherapy, and one with radiation therapy. Two patients were treated with afatinib. One patient received both mobocertinib and amivantamab. Three patients had unknown treatment history.

With an average follow-up time of 24 months (ranging from 0 to 69 months) after initial diagnosis, 17 of the 18 patients developed distant metastases (stage IV). One patient had mediastinal lymphadenopathy but no known distant metastasis after 12 months (Case #8). The most frequent site of metastasis was the brain (9 of 17; 52.9%) followed by the bone and intrapulmonary site (each 7 of 17; 41.1%). Additional sites of metastasis, which were biopsy-proven or clinically suspected by imaging, include the lymph node, liver, and adrenal gland (**Table 1**).

**Table 1:** Characteristics of patients with *EGFR/ERRB2* exon 20 insertion/duplication.

Case	Age (years)	Ethnicity	Smoking (pack/year)	Sex	Presenting stage	Progression to stage IV (months)	Site tested	Morphology	Sites of metastasis	Treatment	Time to event (months)	Outcome
1	60–65	Caucasian	0	M	IIb	22	Lung	AdenoCa, acinar predominant	Intrapulmonary, chest wall	Chemo XRT	69	Alive
2	55–60	African American	40	M	IIIa	4	Lung	AdenoCa, acinar predominant	Bone, liver, adrenal, brain	Chemo XRT	13	Hospice
3	40–45	Caucasian	20	M	IV	N/A	Skull	AdenoCa vs. atypical carcinoid	Bone, liver, brain	Chemo, XRT Rovalpituzumab	31	Decd
4	75–80	Caucasian	48	F	IV	N/A	Lung	AdenoCa, lepidic predominant	Intrapulmonary, LN	Chemo Pembro	2	Decd
5	55–60	Caucasian	0	F	IV	N/A	Lymph node	AdenoCa, acinar and micropapillary	Brain, intrapulmonary, LN	XRT Pembro	1	Decd
6	60–65	Caucasian	0	M	IV	N/A	Lymph node	Metastatic adenoCa	Adrenal, bone, brain, LN	Unknown	1	Hospice
7	45–50	Caucasian	22.5	F	Ia	8	Lung	AdenoCa, acinar predominant	Intrapulmonary, LN	Chemo mobocertinib amivantamab	32	Alive
8	75–80	Caucasian	0	F	Ia	N/A	Lung	AdenoCa	None	XRT	12	Alive
9	55–60	Caucasian	0	F	IIa	41	Lung	AdenoCa, lepidic predominant	Bone, intrapulmonary	None	42	Alive
10	75–80	Caucasian	0	M	IV	N/A	Bone	Metastatic adenoCa	Bone, brain	XRT Afatinib	17	Decd
11	70–75	Caucasian	7.5	F	IV	N/A	Liver	Non–small cell lung carcinoma	Liver, brain	Chemo XRT Pembro	66	Alive
12	60–65	Caucasian	5	M	IIa	7	Lymph node	Adenocarcinoma, papillary predominant	Intrapulmonary, LN	Chemo	30	Decd
13	45–50	Caucasian	0	M	IV	N/A	Lymph node	Metastatic adenoCa	Intrapulmonary, bone, multiple other organs	Afatinib	1	Decd
14	80–85	Caucasian	0	F	IV	N/A	Pericardial fluid	AdenoCa of uncertain origin	Intrapulmonary, pericardial fluid	Unknown	4	Decd
15	40–45	Unknown	Unknown	M	Unknown	Unknown	Liver	Metastatic adenoCa	At least liver	Unknown	Unknown	Unknown
16	70–75	African American	15	M	IIb	22	Lung	Invasive mucinous adenoCa	Brain, LN	Chemo, XRT, Pembro	54	Decd
17	70–75	Caucasian	0	F	IV	N/A	Lung	AdenoCa, acinar predominant	Bone, brain	Chemo, XRT Pembro	9	Decd
18	65–70	Caucasian	10	F	IIIb	13	Lymph node	Metastatic adenoCa	Brain, LN	Chemo, XRT Trastuzumab Pembro	44	Hospice

AdenoCa, adenocarcinoma; Chemo, chemotherapy; Decd, deceased; F/M, female/male; LN, lymph node; N/A, not applicable; Pembro, pembrolizumab; XRT, radiation therapy.

### 3.2 *EGFR/ERBB2* ex20 ins/dup variants and co-occurring genomic alternations

**Figure 1:**
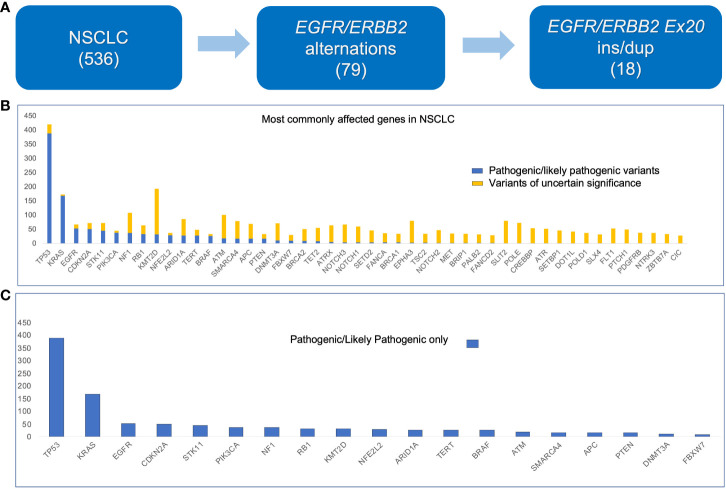
Histogram showing genes with mutations detected in non–small cell lung carcinoma (536 cases) using the 214-gene targeted panel. **(A)** Number of samples used in this study. **(B)** All variants. **(C)** Pathogenic and likely pathogenic variants only.

The variants detected by the 214-gene NGS panel from 536 patients are summarized in **Figure 1**. Seventy-nine patients had *EGFR* or *ERBB2* aberrations; among them 18 patients had ex20 ins/dup (**Figure 1A**). The most frequently mutated gene was *TP53*, followed by *KMT2D* and *KRAS* (**Figure 1B**). Genes that had the most frequent pathogenic/likely pathogenic variants were *TP53*, *KRAS*, and *EGFR* (**Figure 1C**).

**Figure 2:**
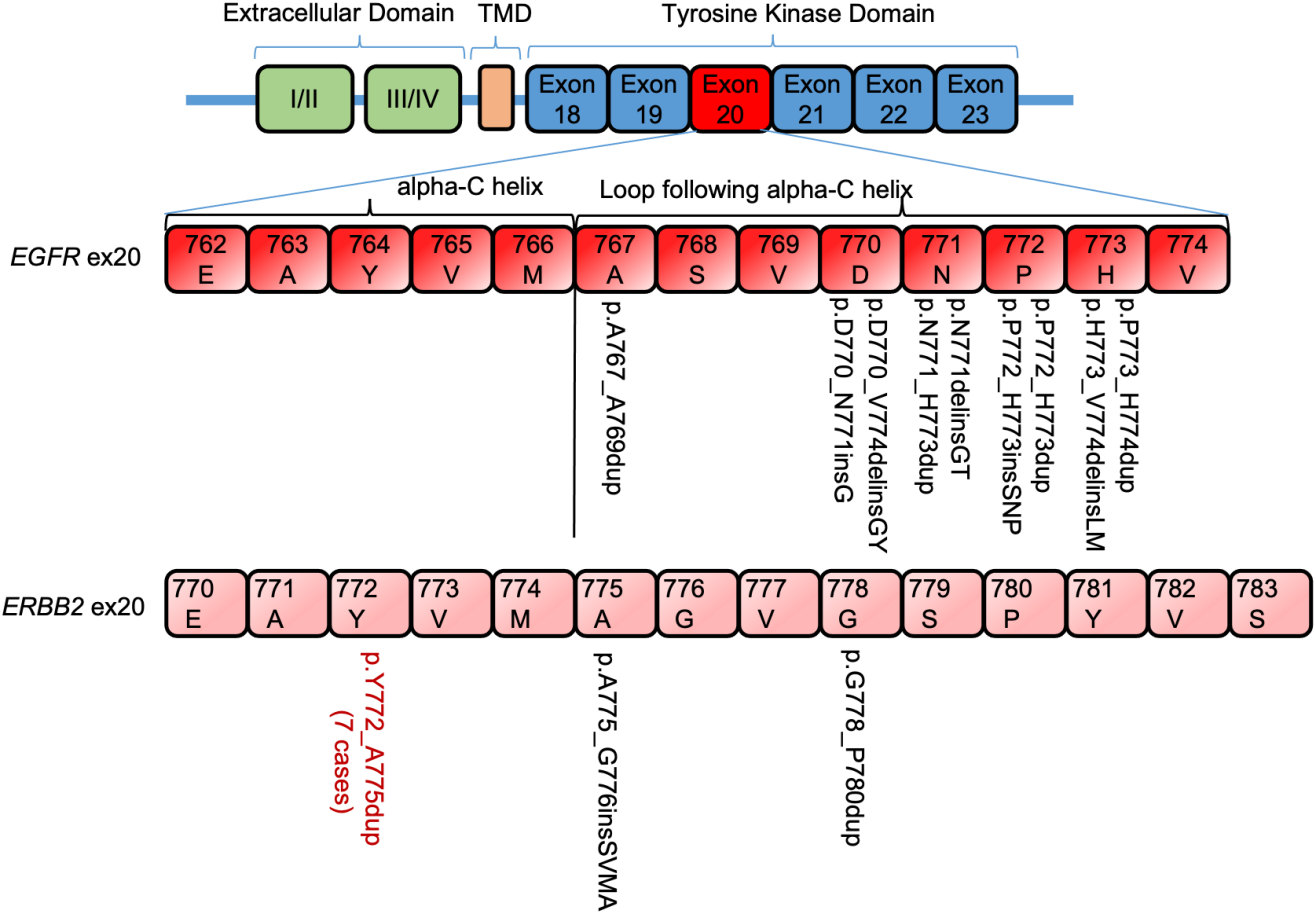
Distribution of *EGFR/ERBB2* exon 20 insertion/duplication variants in non–small cell lung carcinoma. TMD, transmembrane domain.

Nine of each *EGFR* and *ERBB2* ex20 ins/dup variants were identified, which were heterogenous in-frame insertion/duplication of one to four amino acids spanning A767−V774 in *EGFR* and Y772−P780 in *ERBB2*. All the *EFGR* ex20 ins/dup variants were clustered in the loop following the alpha C-helix. Seven of the nine (77.8%) *ERBB2* ex20 dup were duplication of four amino acids (p.Y772_A775dup) in the alpha C-helix domain, and only two (p.A775_G776 insSVMA and p.G778_P780Y) were located in the loop following the alpha C-helix domain (**Figure 2**).

Co-existing variants were identified in 13 (72.2%) *EGFR/ERBB2* ex20 ins/dup-positive cases. *TP53* mutations were the most common co-occurring variants (12 of 18, 67%), followed by *RB1* (3 of 18, 17%). Pathogenic/likely pathogenic variants in *PIK3CA*, *PTEN*, and *CDKN2A* were detected in one case each. In five cases, *EGFR/ERBB2* ex20 ins/dup was the only pathogenic variant. Only Case #9 had a low level of *CDK4* amplification. All other cases had no CNV. No fusion transcript was detected in any cases, and all cases were microsatellite stable (**Table 2**).

**Table 2:** Genomic profiling of *EGFR/ERRB2* Ex20 ins/dup-positive NSCLCs.

Case	Exon 20 ins/dup	Other variants	Copy number variation	PD-L1 (IHC)
1	*EGFR* c.2315_2316insGTCCAACCC: p.P772_H773insSNP	*TP53* c.814G>T: p.V272L *PIK3CA* c.3140A>G: p.H1047R	None	TC : LP IC:N
2	*EGFR* c.2318_2320delinsTCA: p.H773_V774delinsLM	*TP53* c.740_745del: p.N247_R248del	None	TC:N IC:N
3	*EGFR* c.2308_2309insGTT: p.D770delinsGY	None	None	TC:N IC:N
4	*EGFR* c.2310_2311insGGG: p.D770_N771insG	*TP53* c.841G>A: p.D281N 12	None	TPS:N IC:N
5	*EGFR* c.2315_2316insGCACAACCC: p.N771_H773dup	*TP53* c.569C>T: p.P190L	None	TC:P IC:N
6	*EGFR* c.2311_2312delAAinsGGCAC: p.N771delinsGT	*TP53* c.659A>G:p.Y220C	None	TPS:N IC:N
7	*EGFR* c.2300_2308dupCCAGCGTGG: p.A767_V769dup	*TP53* c.915delG: p.K305fs *PTEN* c.367C>T: p.H123Y *PTEN* c.302T>A: p.I101N	None	TPS: N IC: N
8	*EGFR* c.2316_2321dupCCACGT: p.H773_V774dup	*TP53* c.711G>A: p. M237I *RB1* c.2520 + 5G>A	None	TPS: P, IC: P
9	*EGFR* c.2314_2319dupCCCCAC: p.P772_H773dup	*TP53* c.711G>A: p.M237I	*CDK4* gain	TPS: LP, IC: P
10	*ERBB2* c.2313_2324dup: p.Y772_A775dup	None	None	TC: LP IC: N
11	*ERBB2* c.2313_2324dupATACGTGATGGC: p.Y772_A775dup	None	None	TC: N IC: N
12	*ERBB2* c.2313_2324dup: p.Y772_A775dup	*TP53* c.661G>T: p.E221Ter	None	TC: LP IC: N
13	*ERBB2* c.2313_2324dup: p.Y772_A775dup	None	None	TC:N IC:C/A
14	*ERBB2* c.2313_2324dupATACGTGATGGC: p.Y772_A775dup	None	None	TPS:N IC: NA
15	*ERBB2* c.2313_2324dupATACGTGATGGC: p.Y772_A775dup	*TP53* c.652dupG: p.V218Gfs* RB1 c.446C>G: p.S149Ter	None	TPS:N IC:N
16	*ERBB2* c.2313_2324dup: p.Y772_A775dup	*TP53* c.469G>T: p.V157F *CDKN2A* c.307_317del: p.R103fs	None	TPS:N IC:N
17	*ERBB2* c.2331_2339dupGGGCTCCCC: p.G778_P780dup	*TP53* c.659A>C: pY220S	None	TPS:N IC:N
18	*ERBB2* c.2325_2326insTCCGTGATGGCT: p.A775_G776insSVMA	*RB1* c.2243A>C: p.Glu748Ala	None	TC:N IC:N

C/A, cannot be assessed; IC, immune cell; LP, low positive; N, negative; P, positive; TC, tumor cell; TPS, tumor proportion score.

Tumor mutation burden (TMB) information was available for four patients who had molecular tests performed at other facilities. Of these, three cases had low TMB (<10 mutations/mega bases). The only case that had likely high TMB (16 mutations/mega bases, Case #17) was from a patient who was a non-smoker and had negative TPS and IC scores in the tumor.

### 3.3 Molecular and morphologic correlations

Primary tumors were available in 11 *EGFR/ERBB2* ex20 ins/dup-positive cases. Seven cases (64%) had an acinar predominant growth pattern, two (18%) were of the lepidic predominant pattern, and one each had a papillary or mucinous predominant pattern (9%).

### 3.4 PD-L1 immunohistochemistry and correlation with molecular findings

PD-L1 IHC was performed on all ex20 ins/dup-positive cases. TPS and IC were positive in two cases (including one with low positive TPS) and negative in six cases (22C3 clone). TC was positive in one case, low positive in three cases, and negative in six cases. All cases were IC negative (E1L3N clone) (**Table 1**). Among the cases with positive TPS or TC scores, only the one patient with a high TC score was treated with ICI (pembrolizumab) in addition to radiotherapy. This patient had an OS of 1 month (Case #5). Mobocertinib and amivantamab were used as part of the treatment regime for Patient #7, and the patient is still alive 35 months after initial diagnosis.

## 4 Discussion

In-frame insertion/duplication in *EGFR* ex20 comprises 4%–12% of all *EGFR* mutations in NSCLC (14–16) and represents a distinct subset of *EGFR-*mutated NSCLC. Ex20 ins/dup confers resistance to the conventional EGFR TKIs as well as other commonly available immunotherapies (17) and is associated with poor prognosis. The FDA approval of mobocertinib and amivantamab has changed the course for patients whose tumors harbor *EGFR*/*ERBB2* ex20 ins/dup. However, because of the low frequency of ex20 ins/dup in NSCLC, this subgroup of tumors has not been well characterized. Here, we presented findings from the comprehensive genomic profiling of 18 *EGFR* and *ERBB2* ex20 ins/dup-positive NSCLCs including co-existing pathogenic/pathogenic variants, CNV, MSI, and PD-L1 status and correlation of the genomic findings with clinicopathologic features.

Similar to the classic *EGFR* variants, *EGFR* ex20 ins/dup was found more common in Asian women (18), non- or light smokers, and adenocarcinomas (2). In our cohort, 15 patients were Caucasian and two were African American. No Asian ethnicity was identified. All 18 *EGFR/ERBB2* ex20 ins/dup-positive cases were adenocarcinomas and most frequently had the acinar predominant growth pattern (64%, n = 11). Other growth patterns were also identified in ex20 ins/dup-positive cases including two lepidic and one each papillary and mucinous predominant pattern. Fourteen of the 18 patients (78%) were either non-smokers (50%) or light smokers (28%). The most common metastatic site in our patients was the brain (52.9%, n = 18) followed by the lung and bone (33% each), similar to the previous reports (5). In our study, the distribution of *EGFR/ERBB2* ex20 ins/dup variants showed no gender preference. The predominance in Caucasian patients could be due to the bias in our patient population.

*EGFR* and *ERBB2* ex20 ins/dup was each identified in 1.7% of 536 NSCLCs analyzed, similar to the incidence reported in the literature (4, 7). *EGFR* ex20 ins/dup is known to be mostly in-frame insertion/duplication of one to seven amino acids between codons A767 and V774 (8). The V769_D770 and D770_N771 were the two most commonly affected locations and together accounted for approximately 40% of all ex20 ins/dup (4). All the *EGFR* ex20 ins/dup identified in our patients were one to three amino acids ins/dup and located in A767–V774 (**Table 2**). These variants had notable heterogeneity, and each case had a different ex20 ins/dup variant. The p.P772_H773insSNP detected in a male non-smoker patient is novel (Cosmic Database). p.D770delinsGY was reported to have a more favorable response to *EGFR* TKIs (19). This variant was detected in Patient #3, a 41-year-old man who presented with stage IV disease. He did not receive any EGFR-specific TKI and was deceased 31 months after diagnosis.

*ERBB2* and *EGFR* both belong to the ErbB family of receptor tyrosine kinase. The structural analog of ex20 ins/dup that promotes ligand-independent activation of *EGFR* signaling pathway has also been shown in *ERBB2* (14, 19). Similar to the classic *EGFR* TKIs, trastuzumab did not show any clinical benefit against NSCLCs with *ERBB2* ex20 ins/dup (20). *ERBB2* ex20 ins/dup, although uncommon, represents the most common *ERBB2* mutations in NSCLC (14). The most common *ERBB2* ex20 ins/dup was reported to be the p.A775_G776insYVMA in the loop following the alpha C-helix domain (3). Only one patient in our cohort had a variant in this location (p.A775_G776 insSVMA), another case had a variant in the adjacent codon (p.G778_P780Y), and the remaining seven (78%, n = 9) were duplication of four amino acids (p.Y772_A775dup) in the alpha C-helix domain (**Figure 2**).

It has been determined that the mechanism underlying the TKI resistance of *EGFR* ex20 ins/dup is through inducing structural changes (21). Ex20 ins/dup variants are clustered in the alpha C-helix and the P-loop of the EGFR protein that are the key regulatory regions for EGFR activation status (22). Structural alterations in these regions prevent binding of the reversible TKIs to EGFR protein resulting in resistance. Mobocertinib irreversibly binds to *EGFR* with exon 20 insertion mutation, preventing *EGFR*-mediated signaling and leading to cell death. *ERBB2* ex20 ins/dup was targeted similarly (10, 21, 23, 24). The correlation of different variants with metastasis, prognosis, and therapy response was controversial (8, 15, 25).

Almost all *EGFR/ERBB2* ex20 ins/dup variants were mutually exclusive with other known oncogenic drivers in NSCLC such as *KRAS*, *ALK*, or *ROS1* fusions (2). Co-occurring mutations have been reported in NSCLCs with *EGFR* ex20 ins/dup and predominantly were alternations in tumor suppressors such as *TP53* (up to 65%) and *RB1* (11%) and cell cycle inhibitors (cyclin-dependent kinase inhibitor 2A and 2B, 22% and 16%, respectively) (4). Five of the 18 patients with *EGFR*/*ERBB2* ex20 ins/dup had no other mutations providing more evidence that *EGFR/ERBB2* ex20 ins/dup was the oncogenic driver in this molecular subtype of NSCLC. Mutations in *TP53* (67%) and *RB1* (17%) were the most frequently co-occurring aberrations in our cohort of ex20 ins/dup-positive cases. *PTEN* and *CDKN2A* mutation was identified in one case each. *TP53* co-mutation decreased the EGFR TKI efficacy in patients with non-ex20 ins/dup mutated NSCLC (26). The effect of *TP53/RB1* mutation in ex20 ins/dup-positive tumors is uncertain, but the high incidence of co-existing mutations in tumor suppressors may contribute to chemoradiation resistance and poor prognosis of this subgroup of NSCLC.

Riess et al. (2) reported a high incidence of co-occurring *EGFR* amplification in *EGFR* ex20 ins/dup-positive NSCLCs (58 of 263, 22%). In our cohort, only one case had a low level *CDK4* amplification (Case #9). The discrepancy in *EGFR* CNV could be due to the relatively small number of cases in our study.

Immune checkpoint inhibitors have been shown to be ineffective against NSCLC with *EGFR* ex20 ins (2, 4). The study performed by Reiss et al. (2) was the largest and likely the only study that evaluated TMB in *EGFR* ex20 ins/dup-positive cases. They found that *EGFR* ex20 ins-positive NSCLCs with high TMB, which is more common in smoking-associated cases, had a higher response rate to ICIs, potentially due to increased T-cell activity against neoantigens generated by tumor mutations. Less than 4% (n = 263) of their cases had intermediate TMB and only 0.7% (2/263) had high TMB (2). Four of our patients had TMB information available (three low and one likely high). Our patients showed a limited response to ICI even when there was positive PD-L1. One patient (Case #17) has a likely high TMB. The patient received therapy including ICI and had an OS of 9 months. Another patient who had an NSCLC with high TC score was treated with ICI (pembrolizumab) in addition to radiotherapy and had an OS of 1 month (Case #5). It seems that TMB status and the high TC score did not provide significant benefits for these two patients. Large-scale studies are needed to further evaluate immunotherapy response in *EGFR/ERBB2* ex20 ins/dup-positive patients.

**Figure 3:**
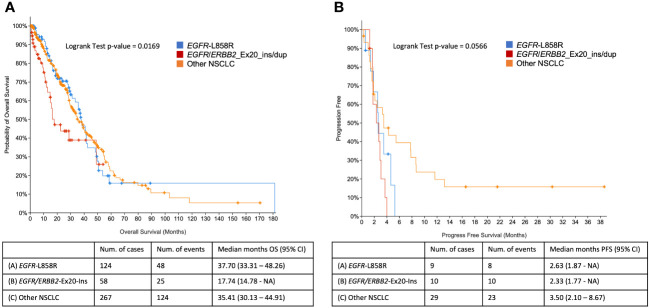
Comparison of overall survival and progression-free survival curves in patients with NSCLC with *EGFR/ERBB2* p.L858R, ex20 ins/dup, and other *EGFR/ERBB2* aberrations using TCGA and MSKCC data in cBioPortal. **(A)** Overall survival. **(B)** Progression-free survival.

Ex20 ins/dup is known to be associated with a worse prognosis compared to other *EGFR* mutations. Seventeen of the 18 patients in our study developed stage IV disease during follow-up (average 24 months). We only had 17 patients (one patient with no clinical information available), and some of the patients are still alive. The small number of patients may not generate meaningful/reliable survival data due to limited statistical power. Therefore, a larger cohort, The Cancer Genome Atlas (TCGA) and Memorial Sloan Kettering Cancer Center (MSKCC) data, was used for survival analysis. Although both ex19 del and p.L858R are associated with better outcome in comparison to ex20 ins/dup, further stratification of the two classic variants showed that patients with NSCLC harboring the p.L858R variant had worse OS compared to patients with ex19 del (27–29). We therefore compared the differences between NSCLC cases with *EGFR/ERBB2* ex20 ins/dup and p.L858R mutation using the TCGA and MSKCC data in cBioPortal. Of 2,743 patients with NSCLC, 573 had *EGFR* or *ERBB2* alterations; of which 73 have *EGFR/ERBB* ex20 ins/dup. Survival analyses showed that patients with *EGFR/ERBB2* ex20 ins/dup had a poor OS compared to patients with *EGFR* p.L858R mutation (*p* = 0.017). The median OS of patients with ex20 ins/dup was 17.7 months, whereas, in patients with *EGFR* p.L858R and other variants, the OS was 37.7 and 35.4 months, respectively. Ex20 ins/dup was also associated with decreased PSF that was 2.33 months in patients with ex20 ins/dup, 2.63 months in patients with p.L858R, and 3.5 months in patients with other *EGFR/ERBB2* mutations (p = 0.057) (**Figure 3**). The lack of statistical significance in PFS could be due to the small number of patients in each group.

In conclusion, we performed extensive studies of NSCLCs harboring *EGFR/ERBB2* ex20 ins/dup. No ethnicity or gender preference was observed. Ex20 ins/dup was frequently associated with non- or light smoking history, adenocarcinomas with acinar predominant growth pattern, negativity for PD-L1, and advanced disease. Our data also suggested that ex20 ins/dup could be sufficient to drive oncogenesis in this molecular subset of NSCLC. Given the notable heterogeneity of these variants and the high rate of co-occurring *TP53/RB1* mutations, additional studies are needed to evaluate the correlation of different *EGFR/ERBB2* ex20 ins/dup variants and co-mutation with targeted therapy. The possible development of resistant mutation after mobocertinib treatment also warrants further investigation.

## Data availability statement

The datasets presented in this article are not readily available because of privacy restrictions. Requests to access the datasets should be directed to the corresponding author.

## Ethics statement

The studies involving human/animal participants were reviewed and approved by the Institutional Review Board on Human Subjects Office of the University of Iowa. Written informed consent from the patients was not required to participate in this study in accordance with the national legislation and the institutional requirements.

## Author contributions

RS performed data search/collection and analysis, put **Figures 1** and **3** together, and revised the manuscript. BD participated in the data collection and drafting of the manuscript, performed chart review, and put the tables together. NG provided technique support for next-generation sequencing and put **Figure 2** together. ADB provided critical review and edits of the manuscript. DM designed the project and participated in drafting, revising, and finalizing the manuscript. All authors contributed to the article and approved the submitted version.
